# Somatic symptoms beyond those generally associated with a whiplash injury are increased in self-reported chronic whiplash. A population-based cross sectional study: the Hordaland Health Study (HUSK)

**DOI:** 10.1186/1471-244X-12-129

**Published:** 2012-08-31

**Authors:** Solbjørg Makalani Myrtveit, Jens Christoffer Skogen, Hanne Gro Wenzel, Arnstein Mykletun

**Affiliations:** 1 Faculty of medicine and dentistry, University of Bergen (UoB), Bergen, Norway; 2 Division of Mental Health, Department of Public Mental Health, Norwegian Institute of Public Health, Bergen, Norway; 3 Research Unit on Mental Health Epidemiology, Department of Health Promotion and Development, Faculty of Psychology, UoB, Bergen, Norway; 4 Division of Psychiatry, St Olav University Hospital, Norwegian University of Science and Technology, Trondheim, Norway; 5 University of New South Wales, School of Psychiatry, Sydney, Australia

**Keywords:** Chronic whiplash, Somatic symptoms, Somatization, Functional somatic syndrome, Biopsychosocial

## Abstract

**Background:**

Chronic whiplash leads to considerable patient suffering and substantial societal costs. There are two competing hypothesis on the etiology of chronic whiplash. The traditional organic hypothesis considers chronic whiplash and related symptoms a result of a specific injury. In opposition is the hypothesis that chronic whiplash is a functional somatic syndrome, and related symptoms a result of society-induced expectations and amplification of symptoms.

According to both hypotheses, patients reporting chronic whiplash are expected to have more neck pain, headache and symptoms of anxiety and depression than the general population. Increased prevalence of somatic symptoms beyond those directly related to a whiplash neck injury is less investigated.

The aim of this study was to test an implication derived from the functional hypothesis: Is the prevalence of somatic symptoms as seen in somatization disorder, beyond symptoms related to a whiplash neck injury, increased in individuals self-reporting chronic whiplash? We further aimed to explore recall bias by comparing the symptom profile displayed by individuals self-reporting chronic whiplash to that among those self-reporting a non-functional injury: fractures of the hand or wrist. We explored symptom load, etiologic origin could not be investigated in this study.

**Methods:**

Data from the Norwegian population-based “Hordaland Health Study” (HUSK, 1997–99); N = 13,986 was employed. Chronic whiplash was self-reported by 403 individuals and fractures by 1,746. Somatization tendency was measured using a list of 17 somatic symptoms arising from different body parts and organ systems, derived from the research criteria for somatization disorder (ICD-10, F45).

**Results:**

Chronic whiplash was associated with an increased level of all 17 somatic symptoms investigated (p<0.05). The association was moderately strong (group difference of 0.60 standard deviation), only partly accounted for by confounding. For self-reported fractures symptoms were only slightly elevated. Recent whiplash was more commonly reported than whiplash-injury a long time ago, and the association of interest weakly increased with time since whiplash (r = 0.016, p = 0.032).

**Conclusions:**

The increased prevalence of somatic symptoms beyond symptoms expected according to the organic injury model for chronic whiplash, challenges the standard injury model for whiplash, and is indicative evidence of chronic whiplash being a functional somatic syndrome.

## Background

The term “whiplash” was first introduced in 1928 [1], describing the lash-like effect caused by sudden acceleration-deceleration forces acting on neck and upper trunk, mainly following a rear impact [2]. A whiplash injury is generally considered an uncomplicated soft tissue injury of the neck, fractures and dislocations are excluded [2].

In the acute phase after a whiplash injury, lasting up to four weeks, the most common symptoms are pain and stiffness of the neck and headache [3,4]. The condition is usually benign and the majority of patients rapidly recover [4-6]. After 3 months, however, recovery seems to level off and despite examinations revealing no neck changes, a substantial amount of patients remains symptomatic [7,8]. Estimates on the transition from acute whiplash to chronic whiplash, defined as symptoms still seen after 6 months [6,9], vary greatly, between 6% and 50% [2,5,10].

Chronic whiplash represents a considerable burden to the society, both health care systems, insurance systems and compensation systems [11]. Patients with chronic whiplash report the disorder to negatively affect their ability to work and reduce their quality of life [2,12]. They also report more symptoms of anxiety and depression than the general population [13,14], and more somatic symptoms like head and neck pain [15-19]. Sufferers of chronic whiplash also report elevated levels of somatic symptoms from body areas not affected by a neck trauma; like gastrointestinal symptoms, palpitations, shortness of breath and sleep disturbances [15,18,20-22].

Some consider chronic whiplash an organic disorder with chronic pain due to injuries in the neck [23], which is what we call the organic hypothesis. There is converging evidence available indicating prevalence of peripheral lesions in some individuals after a whiplash injury [24], but these lesions might not be prerequisite for clinical features seen among sufferers of chronic whiplash [25]. Also, the increased load of diffuse somatic symptoms is difficult to explain if chronic whiplash is considered a mere result of a neck injury. So, regardless if some symptoms are the result of physical injury, it is important to consider other processes than the pure organic that might initiate and maintain symptoms [25].

This has inspired the claim that chronic whiplash is better understood as one of many functional somatic syndromes [21,26,27]. Functional somatic syndromes are characterized by medically unexplained symptoms and suffering [26,28]. The symptoms reported by individuals with functional somatic syndromes are prevalent in healthy populations [29,30] and characterized by diffuse and non-specific symptoms emerging from different organs and body parts [26,28,30-32]. Furthermore, the symptoms of functional somatic syndromes are very similar to somatization disorder, and the two conditions are thought to be closely related [26,33,34].

There are alternative models and explanations for increased symptoms beyond those expected according to the organic model for whiplash. One alternative explanation can be called the recall bias hypothesis. This hypothesis suggests that the generally increased symptom load in self-reported whiplash is a memory, response style or attention bias seen in some individuals, producing positive responses to questions regarding both past injuries and recent symptoms. By way of anxiety, personality, the belief that one is sick, negative expectations about the future course of the disease and stressful events, diffuse symptoms present in the general population might by some individuals be perceived as more noxious and troublesome [26]. Also, individuals experiencing increased symptom load are more likely to think about what could cause their symptoms – and will therefore to a greater extent recall and report injuries of all sorts. This hypothesis can, however, be tested by exploring whether symptoms as seen in somatization disorder are elevated in a self-reported past non-functional disorder, i.e. fractures of hand or wrist.

The purpose of this study was to investigate whether self-reported whiplash injuries are associated with increased prevalence of a broad range of somatic symptoms not readily related to a neck injury. To explore if our finding is merely a result of recall bias, we also explored if the same somatic symptoms are equally increased in self-reported past fractures of hand or wrist. Finally, we aimed to examine if the proportion of the population self-reporting whiplash diminishes with time, and how the symptom burden changes with time since the whiplash injury.

## Methods

### Study population

Data from the “Hordaland Health Study” (HUSK) carried out in Hordaland County in Western Norway between 1997 and 1999 were used. This joint epidemiological research project was conducted in cooperation between the National Norwegian Health Screening Services, the University of Bergen and local health services. The base population included 29,400 individuals born between 1953 and 1957 and aged 40–46 years upon participation. Of these, 18,565 (8,585 men and 9,980 women) accepted the invitation, filled in the questionnaires and attended clinical examinations including measures of blood pressure, height, weight, waist- and hip circumference. This resulted in a general participation rate of 63% (57% for men and 70% for women). For the purpose of the present study, we were interested in individuals reporting whiplash or no whiplash, a fractured hand/wrist or no fractured hand/wrist, and their profile of somatic symptoms. Excluding everyone not answering the questions related to whiplash, a fractured hand/wrist, questions on somatic symptoms and questions on covariates potentially associated with both exposure and outcome, left us with a study population of 13,986 individuals (75% of the HUSK participants; 2,756 excluded due to missing data on outcome and exposure and 1,823 excluded due to missing data on covariates).

### Exposure – whiplash

As in previous studies [13,35], self-reported incident whiplash was assessed using the question “Have you ever experienced whiplash?”, together with a follow-up question on how old the person was at the time of the injury. In correspondence with previous work [21], we defined our group of chronic whiplash sufferers as individuals having experienced the trauma no sooner than one year prior to the survey and reporting neck pain for at least three consecutive months during the last year. Individuals possibly still in their acute phase of the disorder (having experienced whiplash less than one year ago, N = 11) and individuals reporting a whiplash trauma but not having developed chronic neck pain (N = 33) were excluded. This resulted in a group of N = 403 (2.9%) potential sufferers of chronic whiplash. These individuals were compared to the rest of the HUSK participants (no chronic whiplash, N = 13,583) in our analyses.

### Comparison exposure – fractured hand/wrist

In the same section and wording as for whiplash, participants were also asked whether they have ever fractured their hand or wrist. There was no missing for this item and N = 1,746 (12.5%) reported a past fracture of the hand or wrist.

### Outcome - somatic symptoms

The frequencies of common somatic symptoms arising from different body parts and organ systems were investigated. For this, a list of 17 somatic symptoms (abdominal pain, nausea, bloating, tongue plaque, regurgitation, frequent defecation, discomfort in genital region, skin discoloring, joint/muscle pain, dizziness, tiredness, paresthesia in extremities, burning eyes, headache, shortness of breath, chest pain and problems with urination) was used. The list contains 13 items from the ICD-10 research criteria for somatization disorder (F45) [33] and 4 other symptoms related to somatization and functional somatic syndromes [26,28,36]. It has previously been used when exploring somatization [37,38].

The participants were asked to indicate the frequency of which they experience each symptom by ticking off “almost never”, “rarely”, “sometimes”, “often” or “almost always”. The two latter possibilities were truncated to one, yielding a symptom load ranging from 1–4. The mean frequency of complaints was calculated across symptoms for each individual. As in previous studies [37,38], this mean score was used as an indication of somatization tendency.

### Covariates

Functional somatic syndromes, somatic symptoms and self-reported whiplash are related to a whole range of socio-demographic and health related factors [26,27,34,36,39-41]. We have therefore adjusted the association for the following factors:

Gender was registered, as was marital status, grouped into “not married”, “married”, “separated”, “divorced” or “widow/widower”. Socioeconomic status was assessed trough questions on benefit receipt and education. Participants were asked if they, at the time of participation, received any social aid or pension (Table 1). In relation to education, participants were grouped as having completed “compulsory only”, “high school” or “university”. The 133 not answering this question were set to “compulsory only”.

**Table 1:** Summary of the variables included in the analysis, and differences between individuals reporting chronic whiplash and individuals not reporting chronic whiplash

**Covariates**	**Chronic whiplash N = 403**	**No chronic whiplash N = 13,583**	**P-value (Chi-square test)**	**All N = 13,986**
Female	58.3%	53.9%	0.079	54.0%
Anxiety	24.1%	17.6%	0.001	17.8%
Depression	14.6%	9.6%	0.001	9.8%
Sleep quality			<0.001	
* Good*	37.0%	42.6%		42.4%
* Bad*	17.9%	11.1%		11.4%
* Not asked*	45.2%	46.3%		46.3%
Education			0.119	
* Compulsory only*	13.7%	17.5%		17.4%
* High School*	46.9%	45.7%		45.7%
* University*	39.5%	36.8%		36.9%
Benefit recipiency	28.3%	13.0%	<0.001	13.4%
* Sickness benefit*	10.9%	4.6%		4.7%
* Occupational habilitation*	3.5%	1.1%		1.1%
* Disability pension*	11.9%	3.3%		3.6%
* Social benefit*	1.5%	0.5%		0.6%
* Unemployment benefit*	2.5%	1.6%		1.7%
* Dependents’ pension*	0.5%	0.7%		0.7%
* Other*	2.7%	2.6%		2.6%
Physical activity			0.369	
* None*	17.4%	15.5%		15.6%
* Easy*	38.7%	41.9%		41.8%
* Heavy*	43.9%	42.5%		42.6%
Alcohol consumption			0.838	
* No consumption*	27.1%	28.3%		28.3%
* Moderate consumption*	67.5%	66.1%		66.1%
* Heavy consumption*	5.5%	5.6%		5.6%
Daily smoking	37.5%	35.5%	0.416	35.6%
Somatic diagnoses >0	11.9%	8.1%	0.006	8.2%
Marital status			0.090	
* Not married*	11.2%	12.7%		12.6%
* Married*	71.5%	74.6%		74.5%
* Separated*	3.0%	2.4%		2.4%
* Divorced*	13.7%	9.7%		9.8%
* Widow/Widower*	0.7%	0.7%		0.7%
Mean somatisation >2	43.2%	21.4%	<0.001	22.1%

As somatic diagnoses can affect the symptom profile, we are investigating the number of somatic illnesses each individual was suffering from at participation time, or had suffered from earlier. The number of diagnoses was recorded as participants ticked off the following: heart infarction, angina pectoris, stroke, asthma, diabetes or multiple sclerosis. The responses from 18 participants reporting 3 or more somatic diagnosis were truncated to 3.

Anxiety and depression were measured using the “Hospital Anxiety and Depression Scale” (HADS) [42]. HADS is a widely used self-report questionnaire considered reliable for patients in psychiatric and non-psychiatric settings, and for the general population [43-45]. The questionnaire consists of 14 readily understandable questions on symptoms, seven for depression (HADS-D) and seven for anxiety (HADS-A). Somatic symptoms commonly seen in anxiety and depression are excluded, making the scale useful in populations with somatic illness and symptoms. Each item has four alternative responses ranging from symptom not present (0) to maximum reported level of symptom (3), giving a sum score range from 0 to 21 for both subscales. In accordance with previous studies, a valid rating of depression and anxiety was defined as at least 5 completed items on each sub-scale (HADS-S and HADS-D) [45,46], and the recommended cut-off score of ≥8 was used in the descriptive table [43-45].

Health-related behavior was evaluated in line with previous studies [47,48]. The participants were asked “Do you smoke cigarettes, cigars and/or pipe daily” and grouped as smokers or non-smokers. Physical activity was evaluated by asking how often and for how long the participants engaged in both light and intense leisure-time physical activity. Light activity was defined as activity that did not lead to being sweaty or out of breath, while intense activity was activity that did result in sweating or breathlessness. Individuals were then divided into groups performing “no physical activity”, “moderate physical activity” and “heavy physical activity”. Amount of alcohol consumption was assessed using two questions: “Do you abstain from alcohol?” and “What is your normal consumption of alcoholic beverages within 14 days?”. Based on this, using a cut-off value of 15 units, participants were grouped to have “no consumption”, “moderate consumption”, or “high consumption”.

After the first questionnaire and the clinical examination, a second questionnaire was distributed to a random subsample and completed by 8,896 individuals [49]. This questionnaire contained a question on sleep which was included as a covariate in this subsample. Sleep was evaluated by the question “How often do you experience sleep difficulties”, with tick-off possibilities “never/a few times a year”, “once or twice a month”, “once a week” and “more than once a week”. Participants answering one of the two first were considered as having good sleep, participants answering one of the two latter were considered as having bad sleep.

### Statistical procedure

To enable a comparison between the whiplash-group and the no whiplash-group in relation to somatic symptom profiles, the reported frequency of each symptom was standardized (z-scored) with a mean of 0 and a standard deviation of 1 [50]. This standardization enables a straightforward interpretation of reported frequency across the different somatic symptoms. Independent t-tests were then employed to each of the standardized symptoms and the standardized mean frequency of the reported symptoms (somatization).

In order to adjust for covariates, a multiple linear regression model was employed. Each covariate was adjusted for separately, but all regressions included gender. A fully adjusted model was computed. Also a separate regression analysis was conducted, adjusting each symptom for all covariates. These analyses were then repeated for individual self-reporting a past fracture of the hand or wrist.

To investigate the unadjusted association between the standardized mean frequency of reported symptoms and time since whiplash-accident, a linear regression model was employed.

STATA/SE 10 [51] for Mac was used for all analyses.

### Ethics

All the participants in this study gave their written consent upon inclusion. The HUSK study was approved by the Regional Committee for Medical Research Ethics of Western Norway and the Norwegian Data Inspectorate.

## Results

In the analyzed sample (N = 13,986), N = 403 (2.9%) individuals reported chronic whiplash. The individuals in the whiplash group reported more symptoms of anxiety and depression and were more likely to be benefit recipients than those in the comparison group. They also reported a higher number of somatic diagnoses and worse sleep (Table 1).

Compared to the group of individuals reporting no whiplash, the mean reported frequency of somatic symptoms in the whiplash group was 0.60 SD higher. When adjusting for potential covariates, the mean difference in symptom reporting was attenuated to 0.41 SD in the fully adjusted model (Table 2). The variables which separately attenuated the mean difference the most were “benefit receipt”, “anxiety” and “depression”.

**Table 2:** Mean difference of reported somatic symptoms compared between individuals reporting chronic whiplash and individuals not reporting chronic whiplash, and between individuals reporting a past fractured hand or wrist and no previous fractured hand or wrist

**Chronic whiplash compared to no chronic whiplash**			**Fractured hand or wrist compared to no fractured hand or wrist**		
**Variables**	**B**	**(CI)**	**Variables**	**B**	**(CI)**
No adjustments	0.60	0.50-0.69	No adjustments	0.07	0.02-0.12
Gender	0.58	0.48-0.68	Gender	0.12	0.07-0.17
Education	0.59	0.49-0.69	Education	0.13	0.08-0.18
Benefit receipt	0.49	0.40-0.59	Benefit receipt	0.10	0.05-0.15
Anxiety^a^	0.46	0.38-0.55	Anxiety^a^	0.09	0.05-0.14
Depression^a^	0.51	0.42-0.60	Depression^a^	0.11	0.06-0.15
Somatic diagnosis	0.56	0.46-0.66	Somatic diagnosis	0.11	0.07-0.16
Physical activity	0.58	0.48-0.68	Physical activity	0.13	0.08-0.18
Consumption of alcohol	0.58	0.48-0.68	Consumption of alcohol	0.12	0.07-0.17
Daily smoking	0.58	0.48-0.67	Daily smoking	0.12	0.07-0.17
Marital status	0.57	0.47-0.67	Marital status	0.12	0.07-0.17
Fully adjusted	0.41	0.33-0.49	Fully adjusted	0.08	0.04-0.12

Adjustments for covariates; separately and fully adjusted.

^a^Anxiety and depression entered as a continuous variable.

**Figure 1:**
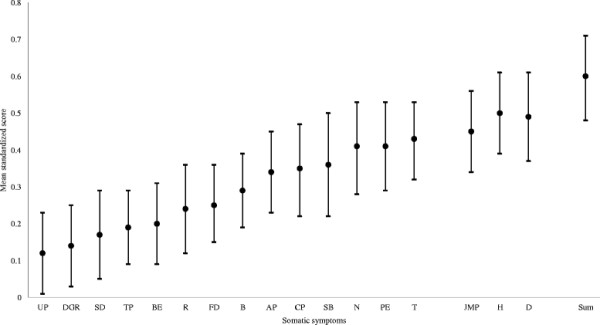
Mean difference in the association between those reporting chronic whiplash and somatic symptoms compared to those not reporting chronic whiplash.

All examined symptoms were elevated in individuals with chronic whiplash compared to those with no chronic whiplash (Figure 1). “Headache” and “dizziness” showed the largest difference in frequency (mean difference 0.50 and 0.49 SD). Also “joint/muscle pain”, “tiredness”, “paresthesia in extremities” and “nausea” had a mean difference at levels of 0.40 SD or higher. There was a mean difference between 0.20 to 0.40 for the symptoms “shortness of breath”, “chest pain”, “abdominal pain”, “bloating”, “frequent defecation”, “regurgitation” and “burning eyes”. The symptoms “tongue plaque”, “skin discoloring”, “discomfort in genital region” and “urination problems” had a mean difference between 0.10 and 0.20 SD. Fully adjusted, all individual symptoms remained statistically significant apart from “urination problems” (p = 0.224) and “discomfort in genital region” (p = 0.159) (Table 3).

**Table 3:** Mean difference of reported frequency of 17 somatic symptoms compared between individuals reporting chronic whiplash and individuals not reporting chronic whiplash and individuals reporting a past fractured hand or wrist and no past fractured hand or wrist, unadjusted, adjusted for gender and fully adjusted

	**Chronic whiplash compared to no chronic whiplash**			**Fractured wrist compared to no fractured hand/wrist**		
**Somatic symptoms**	**Unadjusted B (CI)**	**Adjusted for gender B (CI)**	Fully adjusted* B (CI)	**Unadjusted B (CI)**	**Adjusted for gender B (CI)**	Fully adjusted* B (CI)
Headache (H)	0.50 (0.40-0.60)	0.48 (0.38-0.57)	0.40 (0.31-0.49)	−0.01 (−0.06-0.04)	0.05 (0.01-0.10)	0.04 (−0.00-0.09)
Dizziness (D)	0.49 (0.39-0.59)	0.47 (0.38-0.57)	0.36 (0.26-0.45)	−0.01 (−0.06-0.04)	0.04 (−0.01-0.09)	0.01 (−0.04-0.06)
Joint/muscle pain (JMP)	0.45 (0.35-0.55)	0.44 (0.34-0.53)	0.32 (0.23-0.42)	0.07 (0.02-0.12)	0.11 (0.06-0.16)	0.08 (0.03-0.13)
Abdominal pain (AP)	0.34 (0.24-0.44)	0.33 (0.23-0.43)	0.24 (0.14-0.33)	0.04 (−0.01-0.09)	0.08 (0.03-0.13)	0.05 (0.00-0.10)
Nausea (N)	0.41 (0.31-0.51)	0.39 (0.30-0.49)	0.30 (0.20-0.39)	0.00 (−0.05-0.05)	0.04 (−0.01-0.09)	0.02 (−0.03-0.06)
Bloating (B)	0.29 (0.19-0.39)	0.27 (0.18-0.37)	0.19 (0.10-0.29)	0.02 (−0.03-0.07)	0.08 (0.03-0.13)	0.06 (0.01-0.11)
Tongue plaque (TP)	0.19 (0.09-0.28)	0.18 (0.08-0.28)	0.10 (0.00-0.20)	0.05 (−0.00-0.10)	0.07 (0.01-0.12)	0.04 (−0.01-0.09)
Regurgitation (R)	0.24 (0.14-0.34)	0.24 (0.14-0.34)	0.17 (0.08-0.27)	0.07 (0.02-0.12)	0.06 (0.01-0.11)	0.04 (0.01-0.09)
Frequent defecation (FD)	0.25 (0.16-0.35)	0.26 (0.16-0.36)	0.18 (0.08-0.27)	0.10 (0.05-0.15)	0.07 (0.02-0.12)	0.05 (−0.00-0.10)
Discomfort in genital region (DGR)	0.14 (0.04-0.24)	0.13 (0.03-0.23)	0.07 (−0.03-0.17)	−0.02 (−0.07-0.03)	0.02 (−0.03-0.07)	0.01 (−0.04-0.06)
Skin discoloring (SD)	0.17 (0.07-0.27)	0.17 (0.07-0.27)	0.11 (0.01-0.21)	0.04 (−0.01-0.09)	0.04 (−0.01-0.09)	0.02 (−0.03-0.07)
Tiredness (T)	0.43 (0.33-0.52)	0.41 (0.31-0.51)	0.25 (0.16-0.33)	0.01 (−0.04-0.06)	0.05 (0.01-0.10)	0.01 (−0.03-0.06)
Paresthesia in extremities (PE)	0.41 (0.31-0.51)	0.40 (0.30-0.50)	0.30 (0.20-0.39)	0.04 (−0.01-0.09)	0.06 (0.01-0.11)	0.03 (−0.02-0.08)
Burning eyes (BE)	0.20 (0.10-0.30)	0.19 (0.09-0.29)	0.12 (0.02-0.22)	0.05 (0.00-0.10)	0.08 (0.03-0.13)	0.07 (0.02-0.11)
Shortness of breath (SB)	0.36 (0.26-0.46)	0.36 (0.26-0.46)	0.25 (0.15-0.34)	0.07 (0.02-0.12)	0.08 (0.03-0.13)	0.05 (0.00-0.09)
Chest pain (CP)	0.35 (0.25-0.45)	0.35 (0.26-0.45)	0.26 (0.17-0.36)	0.12 (0.07-0.17)	0.11 (0.06-0.16)	0.08 (0.03-0.13)
Urination problems (UP)	0.12 (0.02-0.22)	0.11 (0.01-0.21)	0.06 (−0.04-0.16)	0.03 (−0.02-0.08)	0.06 (0.01-011)	0.05 (0.00-0.10)

*Adjusted for gender, anxiety, depression, education, benefit receipt, physical activity, marital status, consumption of alcohol, daily smoking and somatic diagnosis.

**Figure 2:**
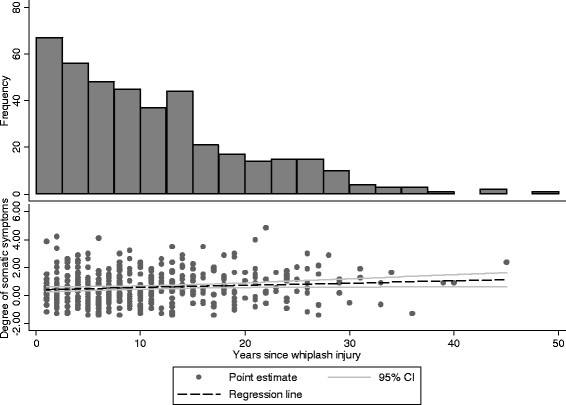
Somatic symptoms, frequency of participants reporting whiplash and time since accident.

The mean time since whiplash injury was 10.8 years (range 1 to 45), and the proportion reporting chronic whiplash declined strongly with increasing time since the whiplash injury (Figure 2). The association between chronic whiplash and somatic symptoms was stronger amongst those reporting having experienced a whiplash injury a long time ago, than amongst those reporting a more resent injury. The tendency was rather weak and borderline significant (within whiplash group, correlation r = 0.016 (p = 0.032) between time since whiplash and somatic symptoms (Figure 2)).

A fracture of the hand or wrist was self-reported by n = 1,756 individuals (12.5%). The mean difference of symptom reporting between those reporting fracture and those reporting no fracture was 0.07 SD. Adjusting for possible confounders changed the mean difference to 0.08 SD. Only six of the somatic symptoms investigated (regurgitation, frequent defecation, joint/muscle pain, burning eyes, chest pain and shortness of breath) showed a significant difference between those reporting a fracture and those reporting no fracture.

## Discussion

### Main findings

Self-report of chronic whiplash was associated with increased frequency of all somatic symptoms examined. Adjusting for potential confounding variables only partly accounted for this difference. A memory or response style bias only cannot explain all of the increased symptom reporting: self-reporting fractures of the wrist or hand was associated with only a few somatic symptoms and only weakly so.

Finally, a declining number of self-reported whiplash injuries were found with increasing time since the accident. Older whiplash injuries were associated with more somatic symptoms reported at participation in HUSK.

### Strengths and limitations

The main strength of this study is the population-based design enabling a comparison between a group of chronic whiplash sufferers and a healthy population. Clinical data would not be appropriate for a study like this due to selection bias. Individuals with a heavy symptom load would to a greater extent seek medical help after a whiplash injury than others, resulting in an exaggerated symptom reporting in the chronic whiplash group. Using a population-based design, we were able to compare all individuals reporting a whiplash injury and neck pain, including individuals not in contact with health services.

The large number of participants and the relatively high participation rate should also be noted as advantages. Furthermore, the dataset contains information on many co-variables, making adjustments for multiple confounders possible. The richness of variables also enabled us to explore if the recall bias hypothesis by comparing our results to that of self-reported fractures.

Both participants in the health study and the staff collecting the data were blinded to the specific hypothesis of this study. Also, the study was conducted in no relation to any litigation, compensation or insurance process. This is important, as such processes have been found to lead to increased symptom reporting and delayed recovery [52,53].

The study also has some notable limitations. Firstly, the study is cross-sectional, making it impossible to conclude on causal inference. For instance, somatic symptoms and symptoms of anxiety and depression might have been present before the accident. Increased pre-injury levels of symptoms of anxiety and depression have been found [35], and reporting low pre-injury physical and mental health predicts whiplash [54].

All data used are self-reported with no objective confirmation. This applies both to self-report of whiplash and fracture of hand or wrist. We do not know if chronic whiplash developed after the whiplash accident: Some individuals in our chronic whiplash group might be recovered from the accident but reporting neck pain with an etiology different from whiplash.

Information based on self-report make the study design vulnerable to possible recall bias; hence increased tendency in some individuals for whatever reason to remember both past injuries and recent symptoms. We aimed to explore the relevance of this bias by repeating all analyses for fractures of the hand or wrist. As symptoms were only slightly increased in individuals self-reporting fractures, we conclude the recall bias problem might be relevant, but cannot entirely explain the increased symptom report in chronic whiplash.

Previous studies have used self-reported data and similar methods of classification when investigating chronic whiplash [13,21]. A recent study found self-reported whiplash to strongly predict a subsequent allowance of disability benefits [35], indicating clinical relevance of self-reported whiplash, whether it is picking up true whiplash sufferers or not. The lack of a medical confirmation is truly a limitation if to be regarded a study of true whiplash victims. We do believe the overlap between self-reported whiplash and sufferers of whiplash injuries who could be clinically verified to be far from perfect, including both false negatives and false positives (i.e. self-diagnosed whiplash). We do, however, also believe that the group self-reporting whiplash is clinically relevant with the highly increased prevalence of somatic symptoms and increased risk of future disability benefits.

Somatic symptoms are also self-reported. The questions related to somatic symptoms were not linked to whiplash in the questionnaire used in HUSK. This could reduce the risk of attribution and symptom accentuating in relation to data collection.

Participants report their symptoms unrelated to any medical condition. We do not know whether the particular participant has an organic explanation for the reported symptoms. Though a broad symptom profile can be difficult to explain within chronic whiplash [55], some of the symptoms seen might have an organic etiology related to the accident. Our study design, did not, however, allow for evaluation of whether or not each symptom was organic in origin.

Also, other somatic diagnoses could lead to increased load of somatic symptoms. Participants were asked if they suffered from heart infarction, angina pectoris, stroke, asthma, diabetes or multiple sclerosis. The association between somatic symptoms and chronic whiplash was adjusted for these comorbid diagnoses. The mean difference in symptom reporting was changed by only 3.4% (from 0.58 to 0.56 SD) when adjusting for these comorbid diagnoses. Whether more of the association could have been explained by inclusion of somatic diagnoses beyond those available in this study remains an open question.

Another limitation is the narrow age span. The base-population for the Hordaland Health Study was 29,400 individuals living in Hordaland County, born between 1953 and 1957. Some researchers find age to hold no prognostic importance after a whiplash accident [56], while others claim older age to increase the risk of poor recovery [57]. A review from 2008 claims conflicting results [58]. Consequently, we cannot exclude the possibility that the narrow age range of our study limits the generalizability of findings.

### Interpretation of findings

Our findings of increased reporting of diffuse symptoms from all body parts among chronic whiplash sufferers and the positive linear association between time since whiplash injury and frequency of somatic symptoms are hard to explain within the organic model. The findings are more compatible with, and indicative evidence of, a functional element within chronic whiplash. However, other explanations may also be relevant.

In line with previous studies [4,7,16,17,19,59-61], the most frequent symptoms amongst chronic whiplash sufferers were headache, dizziness and neck pain/joint-muscle pain. Somatic symptoms beyond headache, dizziness and joint/muscle pain have not previously been as thoroughly explored in the literature. Some studies have found increased levels of gastrointestinal symptoms, palpitations, shortness of breath and sleep disturbances [15,21], but to the best of our knowledge, this is the first study showing that the entire range of somatic symptoms included in the ICD-10 criteria for somatization disorder are increased in individuals self-reporting whiplash.

Different theories aim to explain increased symptom reporting in chronic whiplash: For instance, changes in zygapophysial joints seem to cause neck pain and headache in some individuals after whiplash injuries [24,55,62,63]. In contrast, it is claimed that no MRI changes can be found after whiplash injuries, not for acute [64], nor chronic cases [7,65-67].

Stress system responses including catecholaminergic systems, serotonin systems and the hypothalamic-pituitary-adrenocortical systems also appear capable of producing hyperalgesia and allodynia [25,68].

As in other chronic pain conditions, sensitization might be of importance [25,69]. The sensitization model explains pain as having a physical cause related to changes in the nervous system [70]: After repetitive activation of nociceptors, specific neurons within the spinal cord become sensitized. Also, new connections are made between neurons and inhibitory neurons die. Following this, non-nociceptive stimuli from the periphery may now be misinterpreted as pain. The model further stresses that psychological, behavioral and social problems are related to the existence and persistence of sensitization.

Headache, dizziness and neck pain/joint-muscle pain arise from the neck- and head-area and are therefore the symptoms most easily explained by a whiplash neck injury. As these symptoms are important among individuals suffering from chronic whiplash, we included them in our study. But as they in relation to chronic whiplash cannot be regarded as unexplained diffuse somatic symptoms, they are set aside from the other symptoms in tables/figures.

In our study we cannot explore the cause or origin of symptoms; we solely investigate the symptom load in chronic whiplash. Also, regardless of whether some symptoms are caused by physical injury, other processes might also be of great importance in the development and maintenance of chronic suffering after a whiplash accident.

For instance, theories on symptom amplification and re-attribution [26,40] are useful in explaining our findings. We will consider neck pain as an example. Neck pain is common in the general population, and an individual experiencing a whiplash injury might have had neck pain before the incident. After the accident, however, he/she becomes more aware of the neck pain, and considers the neck pain a result of the injury. The importance of attribution of pre-existing symptoms to the trauma has been emphasized in previous studies [35,71]. In line with this, a tendency to underestimate experienced symptoms such as back pain, neck pain and psychological distress experienced before the accident [72] has been found.

After a whiplash accident, neck pain might also lead to fear of serious damage and chronicity. This again leads to amplification [26,40,73]; neck pain will be more noticed and appear more troublesome.

Looking at this the other way around, individuals experiencing an increased load of somatic symptoms are more likely to spend time thinking about what causes their symptoms. These individuals are therefore more likely than others to remember and report all types of injuries and accidents, which for this purpose can be labeled the recall bias hypothesis. Exploring if somatic symptoms were equally increased in individuals self-reporting a past fracture of hand or wrist, we found at best limited support for this recall bias hypothesis. There was only a very modest increase in the overall somatic symptom score in individuals self-reporting fractures, and limited to only six symptoms.

A declining number of self-reported injuries was found with increasing time since accident. Alongside this, the association between a reported whiplash injury and somatic symptoms got slightly stronger. This finding is contrary to the organic model for chronic whiplash and coherent with several other explanations including the functional model, recall bias, and other explanations.

Anxiety, depression and benefit receipt were the covariates separately attenuating the association between chronic whiplash and somatic symptoms the most. The increased load of symptoms of anxiety and depression found among individuals reporting a whiplash injury, is in line with previous studies [3,13,15,21]. Two explanations have been given for the increased level of anxiety and depression seen in chronic whiplash: It has been considered a psychological response to the injury, like in post-traumatic stress disorder, or as a response to physical pain resulting from the injury [74]. Recent findings do, however, suggest reverse causality, namely that: anxiety and depression at baseline increases the risk of reporting whiplash at follow-up [35]. This debate of cause or effect in the association between whiplash and anxiety/depression does have consequences for whether anxiety/depression is to be regarded a mediating, confounding or even moderating factor in this association. However, our cross sectional design precludes further exploration of the temporal alignment of chronic whiplash and symptoms of anxiety/depression.

The increased symptom reporting, the broad symptom profile and the importance of amplification and attribution, indicate that chronic whiplash cannot merely be considered an organic disorder caused by a neck injury. In previous studies, also other aspects by chronic whiplash have been explored, supporting this.

First, there is a drastically varying prevalence of chronic whiplash in different cultures with similar traffic pattern [40]. Also, the outcome after a whiplash trauma is more affected by cultural expectations [2,75] and cultural factors that generate symptom amplification and attribution [40], than by the actual speed, forces or tissue damage [2,18,76-78]. This is in accordance with the functional model for whiplash, but more difficult to incorporate in relation to the organic model.

Thoughts and emotions in relation to the accident are also of importance for prognosis [78,79]. For instance, the feeling of not being responsible for the accident, and being angry or worried, predict a worse outcome [53,80,81]. Pain-related fear and avoidance appear to be essential in developing chronic pain and disability [73,82]. Also, poor expectations for recovery are tightly associated with poor recovery [83,84].

People experiencing whiplash accidents in relation to sports stand out from other whiplash victims with their absence of chronic symptoms and disability [27]. At the same time, even a placebo rear-end collision without biomechanical potential for injury might give rice to head and neck pain [85]. Finally, individuals self-reporting whiplash have increased risk of being awarded disability pension, also in the absence of neck-pain, and medico-legally for a whole range of diagnoses [35].

The debate over whiplash being a functional or organic disorder is by far settled by this study. But the broad symptom profile found among sufferers of chronic whiplash strongly resembles the diffuse and non-specific profile presented by individuals suffering from functional somatic syndromes [26,28,31,32], and our findings support the repeated suggestions that chronic whiplash is best understood and treated as a functional somatic syndrome [21,26,27].

One attempt to settle or calm the debate over whether chronic whiplash best is regarded an organic disorder or functional somatic syndrome is to introduce alternative perspectives and models. For example, chronic whiplash has been described with a biopsychosocial approach [40]. This alternative model dismisses both the organic and the functional model for whiplash, and suggests that chronic whiplash is a result of cultural expectations, and that symptom reattribution and amplification is of importance. At the same time the possibility of coexisting physical or psychological causes for symptoms is kept open. The biopsychosocial model is broader than the functional somatic model and therefore less readily testable. Though we in our study aimed to investigate the functional somatic syndrome model, our results are also in line with the biopsychosocial model.

### Clinical implications

The debate between the organic and functional model for whiplash has strong clinical implications, but will obviously not be settled on the basis of this study alone. Standard treatment of acute whiplash has been providing the patient with information on injury mechanisms (according to the organic model), advice on suitable activities, recommendations to rest the neck the first weeks, instructions on postural correction, and information on comfort and prevention from excessive movement of the neck that soft collars can provide [86]. Now, studies have concluded that active treatment is more effective [4,86-88] - the patient should be encouraged to do neck exercises [86] and continue if possible to be physically active [27,89]. Clinicians emphasizing the functional somatic syndrome model will try to avoid chronic whiplash by discouraging the patient from assuming the sick-role [26,27], and by undercutting alarming expectations about clinical course [26,73].

## Conclusion

Individuals reporting chronic whiplash also report increased levels of 17 symptoms – including somatic symptoms not readily related to a neck injury. Though an organic origin of these symptoms cannot be excluded in this study, we have difficulties explaining the findings according to the organic disorder model for chronic whiplash. In our opinion, our findings are more in line with the predictions according to the functional somatic model for chronic whiplash. Our findings are also coherent with other etiological models and explanations, including the biopsychosocial model.

## Competing interests

The authors declare that they have no competing interests.

## Authors’ contributions

Authors SMM, JCS and AM designed the study, while HGW contributed further to the scope of the study. SMM conducted literature searches, and provided summaries of previous research. SMM conducted the statistical analysis under the supervision of JCS and AM. All of the authors contributed to the interpretation of the findings, and SMM wrote the first draft of the manuscript. All authors contributed to further refinement of the first draft and the revised manuscript. All authors read and approved the final manuscript.

## Pre-publication history

The pre-publication history for this paper can be accessed here:

http://www.biomedcentral.com/1471-244X/12/129/prepub
